# A Good Work

**Published:** 1886-09

**Authors:** 


### *A GOOD WORK.*

The *Anti-Adulteration Journal*, published monthly at Williamsport and Philadelphia, is almost exclusively devoted to the much needed reform indicated by its title, and it cannot fail to do a good work, and should be liberally patronized.

We know of no sneak thieving confidence scheme quite so contemptible as the adulteration of food, yet in this trafficking age, when the almighty dollar is at once master and slave, it is almost impossible to put a stop to it. Wholesome laws, are ingeniously overreached or openly disregarded, and the open market is full of edibles offered for sale under names which are only calculated to deceive. The effectual way to deal with such trash is to *destroy* it, as did the government inspectors at Cologne, the immense butts of adulterated wine, found in store in that celebrated mart, some years ago, and as also the Board of Health of New York City, recently dealt with the watered milk, at the point of its delivery by rail, for home consumption. Let the good work go on say we, and when the *dishonest* find *honesty* the best *paying* policy, they will be likely to turn from the evil of their ways, and some of us will then be able to make a pretty accurate calculation, that the things we eat and drink are what is claimed for them. But it is not so now.

